# Disseminated Coccidioidomycosis and Coccidioidal Meningitis Hospitalization, Texas, USA, 2016–2023^1^

**DOI:** 10.3201/eid3206.251469

**Published:** 2026-06

**Authors:** Chun Ho Szeto, Alfredo Chavez-Morales, John Garza, Fariba Donovan

**Affiliations:** Texas Tech University Health Science Center Permian Basin, Odessa, Texas, USA (C.H. Szeto, A. Chavez-Morales, J. Garza); University of Arizona College of Medicine–Tucson, Tucson, Arizona, USA (F. Donovan); BIO5 Institute, University of Arizona, Tucson (F. Donovan)

**Keywords:** fungi, meningitis/encephalitis, disseminated fungal infection, coccidioidomycosis, coccidioidomycosis of the meninges, Texas, United States

## Abstract

We analyzed inpatient data from Texas, USA, to characterize hospitalizations for disseminated coccidioidomycosis and coccidioidal meningitis from 2016–2023. Geographic mapping revealed a substantial disease effect in both western Texas and metropolitan areas. Our findings suggest the need for enhanced surveillance and increased healthcare provider awareness regarding coccidioidomycosis in Texas.

Coccidioidomycosis is a fungal infection caused by *Coccidioides* spp. Approximately 1% of all coccidioidomycosis progress to extrapulmonary disseminated coccidioidomycosis (DCM), with involvement of the central nervous system leading to coccidioidal meningitis (CM), the most severe and potentially fatal form of the disease (*1*,*2*).

Coccidioidomycosis is endemic to the southwestern United States, including Texas, where it is not currently a reportable disease (*3**,**4*). During 2016–2021, ≈3,200 coccidioidomycosis-related hospital discharges were recorded in Texas (*5*). Although previous reports have described DCM and CM cases at individual medical centers, state level data for Texas are lacking (*5*,*6*). Our study describes the epidemiology, clinical characteristics, and outcomes of DCM and CM hospitalizations in Texas.

## The Study

We obtained data from Texas Inpatient Public Use Data files (TIPUDF) for January 1, 2016–December 31, 2023, from the Texas Department of State Health Services Center for Health Statistics (https://www.dshs.texas.gov/center-health-statistics). Because the study used publicly available and deidentified data, it was exempt from institutional review board approval. TIPUDF includes claims for medical services from all state-licensed, nonfederal hospitals in Texas, capturing ≈97% of hospital discharges. Each record represents a hospital encounter, containing the final discharge and all related claims information for a deidentified patient.

**Figure 1:**
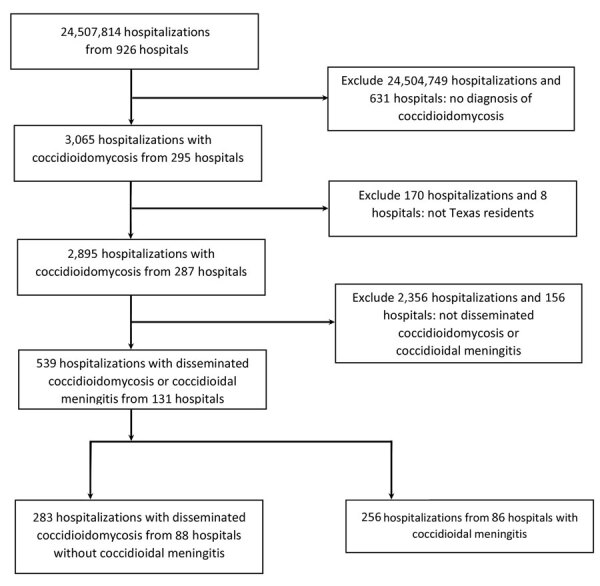
Cohort derivation flowchart for study of disseminated coccidioidomycosis hospitalization, with or without coccidioidal meningitis, Texas, USA, 2016–2023.

We identified the main cohort of DCM on the basis of the presence of codes from the International Classification of Diseases, 10th Revision (ICD-10), related to DCM (B38.7) or CM (B38.4) in all available patient records, including principal and other diagnosis fields (Figure 1). We restricted the analytic sample according to the patients’ US state of residence in record and included only patients with a residence in Texas. This inclusion enabled us to characterize coccidioidomycosis within the Texas population and inform state-specific public health surveillance. 

We divided the cohort into CM and non-CM DCM groups. We identified underlying conditions by using ICD-10 codes (Appendix Tables 1, 2). We identified hospitalizations with intensive care unit (ICU) admissions on the basis of hospital charges coded for an ICU or coronary care unit. We adjusted hospital charges, including facility fee, professional services, pharmacy, laboratory, and all other hospital-billed services, to 2023 Q4 dollars by using the Consumer Price Index for All Urban Consumers obtained from the US Bureau of Labor Statistics (https://www.bls.gov/news.release/archives/cpi_01112024.htm). We based geographic distribution analyses and mapping on patient county of residence. We used the Fisher exact test for comparison of categorical variables and Student t-test for comparison of continuous variables and considered 2-sided p<0.05 statistically significant. We conducted all analyses by using R version 4.4.1 (The R Project for Statistical Computing, https://www.r-project.org). This study is reported in accordance with the RECORD guidelines for observational studies (https://www.record-statement.org) by using routinely collected health data.

For 2016–2023, we identified 539 DCM associated hospitalizations in Texas, USA (Appendix Table 3). Of those, 47.5% (n = 256) involved CM, and 52.5% (n = 283) were DCM without CM. Men represented 59.2% and women 40.8% of hospitalizations. The most affected age group was 18–44 years of age (43.4%), followed by 45–64 years (36.0%). Hispanic patients represented 51.9% of the hospitalizations, followed by Black patients (20.0%).

Pulmonary coccidioidomycosis was documented in 12.6% of DCM hospitalizations. The most common underlying conditions were malnutrition (27.8%), diabetes mellitus (27.3%), and nicotine dependence (23.6%). HIV infection was noted in 15.2% and renal disease in 20.8% of hospitalizations. ICU admission was required in 50.1% of hospitalizations. The mean length of stay was 14.6 days, and the mean adjusted hospitalization charges were $190,933 per stay. In-hospital mortality occurred in 5.9% of hospitalizations, and 4.6% of patients were discharged to hospice.

**Figure 2:**
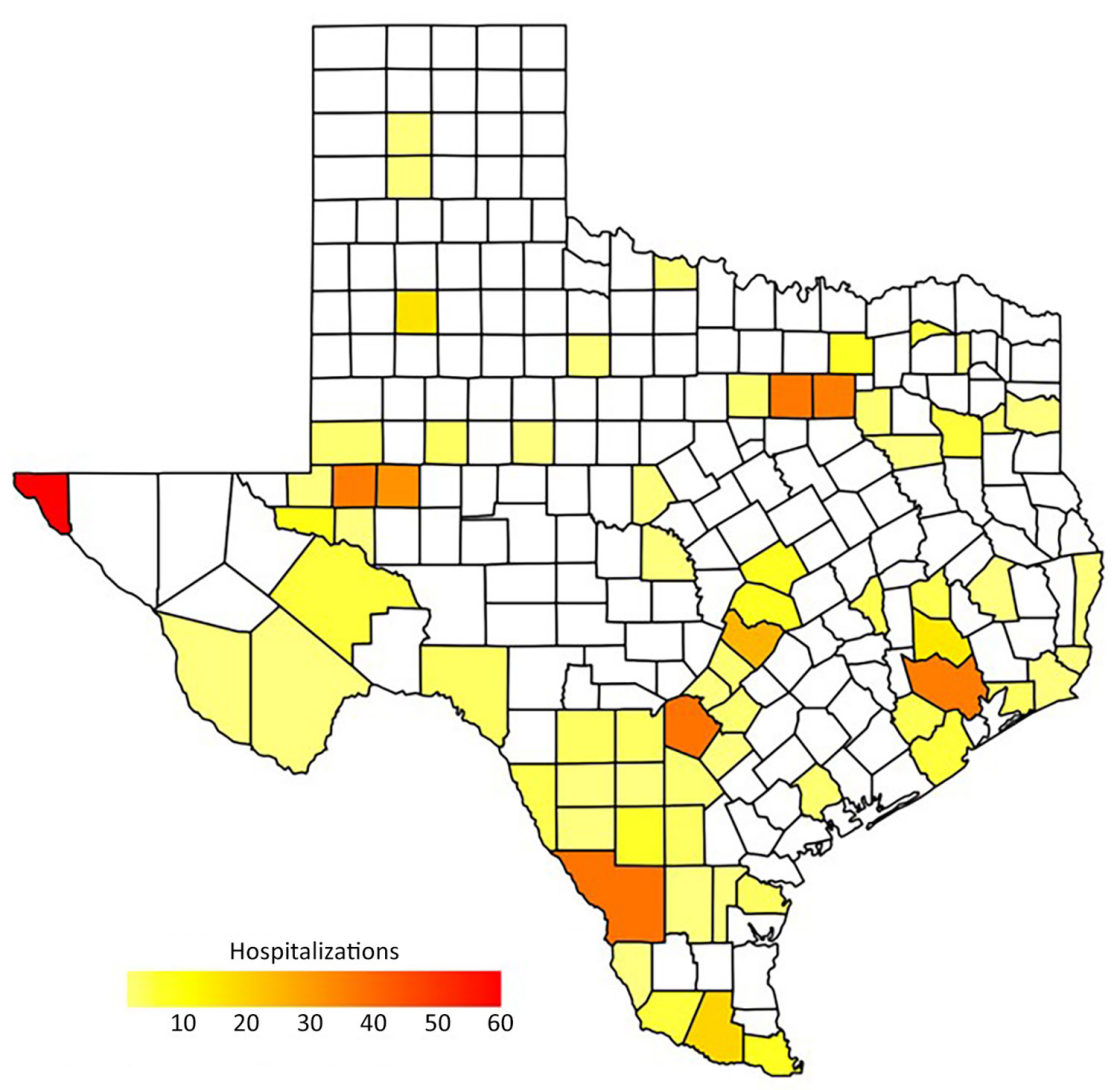
Geographic distribution of hospital visits for disseminated coccidioidomycosis, with and without coccidioidal meningitis, on the county level, Texas, USA, 2016–2023. All counties of Texas with reported cases (n = 96) were included in the study.

The highest rate of DCM hospitalizations occurred in the Odessa–San Angelo region (15.18 hospitalizations/100,000 population), followed by El Paso (6.98 hospitalizations/100,000 population) and Corpus Christi–Laredo (3.34 hospitalizations/100,000 population) (Table). County-level mapping revealed clustering in west-central, south, and east Texas; several counties reported >40 hospitalizations (Figure 2). Hospitalizations for CM and non-CM DCM showed similar patterns in age, gender, and outcomes, but CM hospitalizations showed lower rates of diabetes mellitus, malnutrition, and renal disease.

**Table:** Geographic distribution of disseminated coccidiomycosis and coccidioidal meningitis associated hospitalizations in Texas, 2016–2023

Public health region*	No. hospitalizations in the region/population in the region†	Crude hospitalization rate per 100,000 population (95% CI)
Odessa-San Angelo (9)	93/612,773	15.18 (12.25–18.59)
El Paso (10)	62/888,720	6.98 (5.35–8.94)
Corpus Christi-Laredo (11)	75/2,246,397	3.34 (2.63–4.19)
San Antonio (8)	63/3,026,095	2.08 (1.60–2.66)
Amarillo–Lubbock (1)	18/900,807	2.00 (1.18–3.16)
Tyler (4)	16/1,149,993	1.39 (0.80–2.26)
Austin-Temple (7)	46/3,661,292	1.26 (0.92–1.68)
Dallas-Fort Worth (3)	88/8,044,641	1.09 (0.88–1.35)
Houston (6)	67/7,297,022	0.92 (0.71–1.17)
Abilene-Wichita Falls (2)	5/549,130	0.91 (0.30–2.12)
Beaumont (5)	4/768,635	0.52 (0.14–1.33)

*Defined by Texas Department of State Health Services (https://www.dshs.texas.gov/center-health-statistics/texas-county-numbers-public-health-regions). Numbers in parentheses represent region assignment. †Defined by the US Census in 2020, data provided by Texas Demographic Center (https://demographics.texas.gov/Projections/2024)

## Conclusions

Our study characterizes the geographic distribution and clinical effect of DCM and CM hospitalizations in Texas. Approximately 46% of hospitalizations involved residents of traditionally endemic regions (*5*,*7*). The Odessa–San Angelo region, despite its small population, had the highest rate of DCM and CM hospitalizations, likely reflecting its suitable *Coccidioides* habitat and oil and gas industry activities involving soil disturbance (*7*) (https://www.eia.gov/petroleum/drilling). We observed high absolute numbers of hospitalizations among residents of metropolitan areas, such as Dallas–Fort Worth and Houston, which are not recognized as endemic regions. That observation might reflect the large metropolitan population bases but could also indicate endemic region expansion in Texas related to increased temperatures and altered precipitation patterns (*5*). Travel to endemic regions, both in-state or out-of-state, might also contribute to the observed distribution. However, our dataset lacks travel history information, preventing distinction between locally acquired and travel-related infections. 

The predominance of working-age men in our study is consistent with other publications (*8*,*9*) and likely reflects occupational exposure in construction, agriculture, and oil and gas extraction (*10*,*11*). We also observed a high number of DCM hospitalizations among Hispanic patients (51.9%) in Texas, likely because they comprise 69.4% of the population in endemic west Texas counties (https://demographics.texas.gov/Projections/2024). Black patients, who comprise only 3.9% of the west Texas endemic region population, were disproportionately affected (20.0%) relative to their regional presence, consistent with previous studies documenting higher risk for DCM in African American populations (*8*) (https://demographics.texas.gov/Projections/2024). CM hospitalizations showed lower rates of diabetes, malnutrition, and renal disease compared with non-CM DCM, although the underlying mechanisms remain unclear.

Our study reveals the mortality, length of stay, and charges of hospitalizations among the DCM hospitalizations in Texas. DCM hospitalizations in Texas carry a large financial weight; >50% of patients are hospitalized for ≈2 weeks, and ≈50% required ICU admission. Comparing those outcomes with previously reported national data is limited by methodological and temporal differences. CM-associated mean hospitalization charges were 3.8-fold higher than the national cost estimate reported previously because of our use of unadjusted charges versus cost-to-charge ratio conversions, rather than true differences in resource utilization (*9*). Comparisons of clinical outcomes are further confounded by differing study periods and COVID-19 pandemic-related healthcare disruptions. 

The first limitation of our study is that the sensitivity and specificity of ICD-10 codes for DCM and CM have not been evaluated. Coding errors might result in underestimation of cases. Recent validation work revealed 38.9% of cases coded as unspecified coccidioidomycosis represented disseminated disease (*12*). Second, TIPUDF suppression rules mask gender for patients with a diagnosis code determined to be of a sensitive nature, such as polysubstance use and HIV. That rule affected ≈21.0% (n = 115) study records, which might influence our results. Third, TIPUDF does not distinguish recurrent admissions of individual patients, resulting in duplicated data and overrepresentation of cases with severe disease requiring multiple visits. Fourth, readmission rates for DCM can reach 59%, particularly for CM (*13*). Therefore, our hospitalization-level mortality estimate of 10.5% likely underestimates true patient-level mortality. Finally, lack of mandatory reporting and limited provider awareness might contribute to underdiagnosis (*14*).

Our findings highlight the clinical and economic effects of DCM and CM hospitalizations in Texas and suggest possible expansion of endemic regions. Further economic analysis is warranted to elucidate the financial hardships of coccidioidomycosis on Texas residents. Enhanced surveillance, provider awareness, and targeted public health interventions are needed to address the evolving epidemiology of coccidioidomycosis in Texas.

Appendix Additional informational about coccidioidomycosis and coccidioidal meningitis hospitalization, Texas, USA, 2016–2023.
